# Microbial Immuno-Communication in Neurodegenerative Diseases

**DOI:** 10.3389/fnins.2017.00151

**Published:** 2017-03-23

**Authors:** Bevan S. Main, Myles R. Minter

**Affiliations:** ^1^Laboratory for Brain Injury and Dementia, Department of Neuroscience, Georgetown University Medical CenterWashington, DC, USA; ^2^Department of Neurobiology, University of ChicagoChicago, IL, USA; ^3^The Microbiome Center, University of ChicagoChicago, IL, USA

**Keywords:** microbiome, immunity, blood brain barrier, blood cerebrospinal fluid-brain barrier, Alzheimer's disease, Parkinson's disease, stroke, neuro-inflammation

## Abstract

Neuro-inflammation is a critical process by which the brain coordinates chemokine-regulated cellular recruitment, cytokine release, and cell-mediated removal of pathogenic material to protect against infection or brain injury. Dysregulation of this immune response is involved in multiple neurodegenerative disorders, however the precise contribution of neuro-inflammation to the exacerbation and progression of these diseases remains unclear. Evidence now suggests that commensal micro-organisms populating the host and their metabolites, collectively termed the microbiome, regulate innate immunity by influencing peripheral immune cell populations, and modulating microglial phenotype. Recent preclinical studies now demonstrate that perturbations in the host microbiome can induce alterations in pathological phenotypes associated with numerous neurodegenerative diseases. How perturbations in the host microbiome and subsequently altered peripheral immune status are communicated to the brain to influence neuro-inflammatory processes in these neurodegenerative disease settings is far from understood. This review provides insight into the regulation of neuro-inflammatory processes by the host microbiome in the context of neurodegenerative disease and highlights the potential importance of the blood-brain barrier and blood-cerebrospinal fluid-brain barrier, functioning as “immune barriers,” to communicate host immune status to the brain. Understanding the mechanisms by which the commensal microbiome communicates with the brain to influence neuro-inflammatory processes will be critical in the development of microbially-targeted therapeutics in the potential treatment of neurodegenerative disorders.

## Introduction

Decades of research investigating neuro-inflammatory processes has supported the involvement of resident surveying CNS cells (microglia/astrocytes), in initiating innate immune responses that contribute to neurodegenerative disease pathology. Coordinated glial reactivity is required for efficient removal of pathogenic insults and cellular debris arising from tissue injury, as dysregulation of this CNS neuro-inflammatory response contributes to the progression of neurodegenerative disorders including traumatic brain injury (TBI) (Karve et al., 2016), stroke (Xiong et al., 2016), multiple sclerosis (MS) (Patejdl et al., 2016), Parkinson's disease (PD) (Taylor et al., 2013), and Alzheimer's disease (AD) (Minter et al., 2016b). Within these neurodegenerative states, microglia exhibit a biased pro-inflammatory activation state and great effort has been made to identify therapeutic means by which to correct this bias, promoting anti-inflammatory and neuroprotective microglial activities (reviewed in, Du et al., 2016). Recent evidence now suggests that complex bilateral communication between the peripheral immune status and central immunity plays a key role in influencing various neuro-inflammatory processes, including microglial activation.

A critical immunological compartment regulating general host immune homeostasis (reviewed in, Honda and Littman, 2016) and microglial development is the microbiome, the diverse commensal microbial community (and their metabolites) that populate host tissues. C57BL/6 mice treated with broad-spectrum combinatorial antibiotics (ABX), to deplete microbiota, or generated in gnotobiotic facilities (Germ-Free, GF) display elevated microglial number, marked hypertrophy and alterations in the transcriptome that governs basal microglial surveillance (Erny et al., 2015). Upon exposure to lipopolysaccharide (LPS) or lymphocytic choriomeningitis virus these microglia secreted less pro-inflammatory cytokines and chemokines, suggesting that GF microglia are either naïve to inflammatory stimuli or contain adaptations that shift their activity toward an anti-inflammatory state. Interestingly, supplementation with microbially-derived short-chain fatty acids (SCFAs) was sufficient to restore the microglial function of GF mice akin to specific-pathogen free (SPF)-housed mice. Using a transcriptomics approach, microglia isolated from GF mice displayed significantly down-regulated expression of gene clusters associated with microglial maturation and innate immune responses, specifically type-1 interferon (IFN) signaling (Matcovitch-Natan et al., 2016). These studies demonstrate an important role of commensal host microbiota in regulating microglial development and homeostasis, hence investigators are now interested in ascertaining the role of the microbiome in brain health and neurodegenerative disease.

## The commensal microbiome in neurodegenerative diseases

The importance of exposure to diverse microbiota, from birth, in promoting healthy neuronal development, and connectivity has long been documented (Kim et al., 2003; Juarez et al., 2008). A recent study now demonstrates that ABX-induced microbial perturbations results in basal and exercise-induced hippocampal neurogenic defects (Mohle et al., 2016). This deficit is attributed to the reduction in infiltrating Ly6C^hi^ monocytes, as adoptive transfer of these cells into ABX-treated mice is sufficient to restore neurogenesis. ABX-induced microbial dysbioisis also worsens recovery after spinal cord injury in mice, resulting in decreased white matter volume and enhanced pro-inflammatory macrophage, CD45R^+^ B-cell and CD3^+^ T-cell infiltration within the lesion epicenter (Kigerl et al., 2016). Interestingly, probiotic supplementation post-spinal cord injury enhances anti-inflammatory FoxP3^+^ T regulatory cell (T-reg) recruitment and protects against white matter deterioration.

The host commensal microbiome also regulates neuronal myelination whereby GF mice exhibit hyper-myelinated axons in the pre-frontal cortex and enhanced expression of myelinating gene clusters at the transcriptomics level (Hoban et al., 2016). MS is a complex autoimmune disorder resulting in progressive demyelination of CNS-residing neurons with sufferers possessing altered gut microbial composition (Chen et al., 2016; Jangi et al., 2016). Strikingly, *Proteobacteria* is readily detected within inflammatory demyelinating lesions of cerebral white matter (Branton et al., 2016). Prophylactic administration of polysaccharide A (PSA), produced by the commensal *Bacteroides fragilis*, alleviates experimental autoimmune encephalitis-induced clinical-like symptoms, protects against demyelination and reduces pro-inflammatory cytokine burden (Wang et al., 2014). These beneficial observations result from PSA-induced expansion of CD39^+^/CD4^+^ T-cell populations through toll-like receptor (TLR)-2 signaling that regulate FoxP3^+^ T-reg function within the cervical and mesenteric lymph nodes. These studies suggest the microbiota influence critical immune compartments and regulate neuronal development, myelination, and recovery after injury. Given this, the field is now focusing on the role of the microbiome in neurodegenerative diseases that exhibit hallmark neuro-inflammatory pathology.

Stroke patients develop a vessel occlusion that deprives downstream tissue of vital nutrients, triggering a primary area of cell death. Upon tissue reperfusion a neuro-inflammatory response is initiated that contributes to a secondary injury termed the penumbra. ABX-treated mice subjected to the middle cerebral artery occlusion (MCAO) model of ischemic stroke display a 60% reduction in brain infarct volume and improved sensorimotor function (Benakis et al., 2016). These mice display elevated levels of FoxP3^+^ T-reg cells that suppress IL-17^+^ γδ T-cell proliferation and trafficking to the meninges post-MCAO. The observed reduction in brain CXCL1 and CXCL2 expression then alleviates inflammatory leukocyte infiltration within the infarct and improves outcome after the ischemic event.

In AD, deposition of amyloid-beta (Aβ) confers a plaque-localized neuro-inflammatory response that contributes to disease progression. A recent study has now correlated specific up-regulated pro-inflammatory gut bacteria with elevated pro-inflammatory mediator expression in clinically diagnosed AD patients (Cattaneo et al., 2016). Interestingly, AD sufferers exhibit increased susceptibility to bacterial infection and patients display plaque deposition co-localized with *E. coli*-derived LPS or K99 pili proteins (Zhan et al., 2016). Furthermore, intra-cerebral injection of viable *Salmonella Typhimurium* triggers seeding and accelerated Aβ deposition in 5xFAD mice (Kumar et al., 2016). These findings were validated in transgenic nematodes and reinforce the known anti-microbial nature of Aβ (Soscia et al., 2010), but potentially highlight a novel mechanism for AD exacerbation. A recent study now provides evidence that GF Thy1-APP_SWE_/PS1_L166P_ mice exhibit reductions in Aβ pathology and microglial reactivity (Harach et al., 2017). Additionally, ABX-induced perturbations in the microbiome of APP_SWE_/PS1_ΔE9_ mice alters peripherally circulating cytokine profiles, confers reductions in Aβ deposition and reduces plaque-localized gliosis (Minter et al., 2016c). Both the aforementioned MCAO and APP_SWE_/PS1_ΔE9_ mouse studies demonstrate that microbial abundance post-ABX treatment recovers rapidly, however community diversity is significantly reduced with both displaying dominant expansion of *Lachnospiraceae* and *Verrucomicrobiaceae*. This implies that it is not necessarily the sheer number of microbiota, but rather their population diversity, that impact on host physiology.

Similar to AD, alpha-synuclein (α-syn) deposition observed in PD patients triggers a deposit-localized neuro-inflammatory response that facilitates disease progression. One un-conventional PD exacerbation hypothesis suggests that aberrant gut-localized accumulation of α-syn propagates to the brain via vagal nerve retrograde transport and stimulates pathogenic α-syn seeding (Burke et al., 2008; Del Tredici and Braak, 2008). Whilst this proposal remains controversial, it is now documented that PD patients harbor distinct alterations in microbial diversity of the gastrointestinal (GI) tract (Scheperjans et al., 2015; Unger et al., 2016). A recent seminal study now demonstrates that GF α-syn overexpressing mice display improved motor function and attenuated α-syn aggregation within the caudate putamen and substantia nigra in comparison to SPF-housed mice (Sampson et al., 2016). These mice display altered microglial morphology and reduced pro-inflammatory TNFα and IL-6 cytokine load and these observations are corroborated in ABX-treated α-syn mice. Supplementation of GF α-syn mice with microbially-derived SCFAs exacerbated motor deficits and importantly, GF mice colonized with fecal matter from PD patients resulted in enhanced motor dysfunction compared to mice that received material from healthy controls.

Similar to Aβ, α-syn possesses anti-microbial activity against multiple bacterial, yeast, and fungal strains (Park et al., 2016). In subsequent AD and PD studies it will be critical to address whether the attenuated Aβ and α-syn deposition observed in microbially-perturbed mouse models is a direct result of less protein seeding, due to a reduction in microbial stimulus, or an altered host immune response that triggers active peptide clearance. Regardless, these studies highlight the importance of the commensal microbiome in regulating host immunity and pathology in multiple neurodegenerative disease settings.

## Addressing microbiome-host interactions: a role for immune barriers?

The aforementioned preclinical studies suggest that altering microbial composition elicits distinct changes in host immune compartments, impacting pathology in neurodegenerative disease models. This may be through direct modulation of microglial development and function within the brain parenchyma or through alteration of peripheral immune cell populations that communicate with the brain. The underlying question remains how a perturbation in the microbiome is communicated to the brain to induce functional changes and confer phenotypic alterations observed in these preclinical studies?

TLR expression on epithelial cells of the GI tract permits the host to constantly survey and react to the luminal microbiome (Mu et al., 2015). This monitoring mechanism ensures gut health by modulating host-immune responses to ensure microbial homeostasis. The vagal nerve innervates both mucosal villi and epithelial layers of the GI tract and can be activated in response to inflammatory mediators, produced from TLR signaling, or microbially-derived metabolites to initiate communication of the peripheral immune state to brain (Forsythe et al., 2014). Subsequent activation of the hypothalamic-pituitary-adrenal axis and the anti-inflammatory cholinergic neuronal reflex in the CNS results in significant alterations of innate immunity (Petra et al., 2015, Figure 1B).

**Figure 1:**
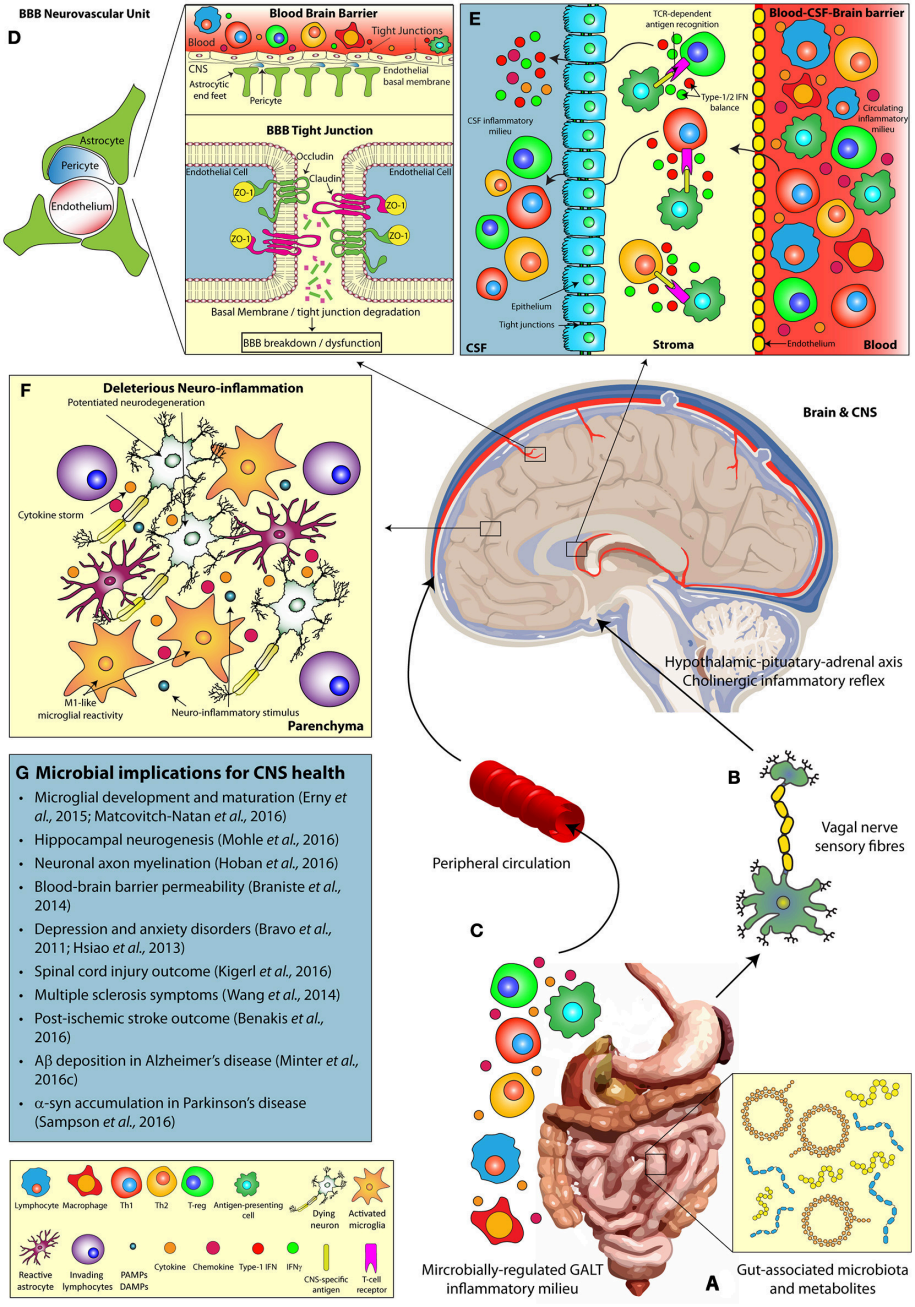
**The microbiomes influence on host CNS health and neurodegenerative disease**. Multiple pathways facilitate the bi-directional communication between the microbiome and the CNS, influencing brain function. **(A)** Alterations in gut microbial composition and metabolomics profiles can induce **(B)** direct stimulation of the vagal nerve, resulting in activation of the hypothalamic-pituitary-adrenal and regulation of innate CNS immune responses. **(C)** In addition, microbially-regulated GALT immune cell populations and the commensal microbiota themselves can alter the circulating peripheral inflammatory milieu (including cytokine profiles), which in turn play a key role in **(D)** the modulation of BBB integrity (via tight junction degradation) and **(E)** T cell infiltration at the BCSFBB. Alterations in the gut microbial diversity may also influence **(F)** deleterious neuro-inflammatory CNS responses, including modulations in microglial reactivity, and cytokine production, **(G)** impacting the progression of neurodegenerative disease states. α-syn, Alpha-synuclein; Aβ, Amyloid-beta; BBB, Blood-brain barrier; BCSFBB, Blood-cerebrospinal fluid-brain barrier; CD, Cluster of differentiation; CNS, Central nervous system; CSF, Cerebrospinal fluid; DAMP, Damage-associated molecular pattern; GALT, Gut-associated lymphoid tissue; IFN, Interferon; PAMP, Pattern-associated molecular pattern; TCR, T-cell receptor; Th, T-helper cell; T-reg, T-regulatory cell; ZO, Zonula occludens.

Vagal nerve activation in response to gut microbial dysbiosis is considered a major contributor to exacerbation of depressive and autism spectrum disorders. Mice populated with *Lactobacillus rhamnosus* displayed differential expression patterns of GABA receptors throughout the brain and attenuated depressive symptoms (Bravo et al., 2011). These beneficial probiotic effects were not observed in vagotomized mice and additional evidence describing similar beneficial effects of *B. fragilis* supplementation in a maternally-immune activated anxiety mouse model supports these findings (Hsiao et al., 2013). Alongside vagal activity an autonomic nervous mechanism conferring gut microbial perturbations following brain injury is also evident (Houlden et al., 2016).

Despite the importance of vagal nerve stimulation in gut-brain axis communication, there remains alternate immune barriers of the brain that are critical in sensing the microbially-regulated circulating peripheral inflammatory milieu (Figure 1C).

### Blood brain barrier (BBB)

The BBB consists of capillary endothelial cells (connected by tight junctions), astrocytes, and pericytes that form the neurovascular unit responsible for barrier integrity (Sweeney et al., 2016). These barrier units separate the CNS and systemic circulation, thus regulating the brain microenvironment independently from the circulating inflammatory milieu. Critically, loss of BBB integrity significantly contributes to the progression of neurodegenerative disorders including TBI (Price et al., 2016), AD (Ryu and Mclarnon, 2009), and PD (Kortekaas et al., 2005). The commensal microbiota regulate peripheral immune cell compartments, implicated in these disorders, and secrete immunologically-active metabolites that circulate to the brain. Thus, the question remains of how the microbiome potentially regulates BBB permeability and stability (Figure 1D) and what implications this has for inflammatory responses in neurodegenerative diseases?

Indeed, gram positive bacterial components elicit pro-inflammatory cytokine responses which alter tight junction protein expression and disrupt BBB integrity *in vitro* (Boveri et al., 2006). Microbial perturbations induced by feeding rodents a “Western diet,” high in saturated fat and sugar (Claesson et al., 2012), triggers increased BBB permeability and cognitive deficits (Davidson et al., 2013). This observation suggests the microbiome may be a previously un-recognized regulator of BBB integrity. Indeed, a seminal study by Braniste et al. demonstrated elevated BBB permeability in fetal and adult GF mice independent of sex (Braniste et al., 2014). This BBB dysfunction was associated with reduced expression of the tight junction proteins occludin and claudin-5. Furthermore, colonization of GF mice with flora from SPF mice restored BBB integrity identified by reduced extravasation of Evans blue dye into brain parenchyma, alongside restored expression of occludin and claudin-5. Interestingly, these results were also replicated when GF mice were mono-colonized with the bacterial strains *Clostridium tyrobutyricum* and *Bacteroides thetaiotaomicron*, both of which produce the SCFAs butyrate, acetate and propionate. Collectively, these data highlight the importance of specific microbial populations, particularly SCFA-producing strains, in regulating BBB physiology.

The therapeutic benefits of butyrate, provided either as pure sodium butyrate or generated by SCFA-producing bacteria, has been recognized in neuropsychiatric disorders (Bourassa et al., 2016), yet the underlying mechanism(s) by which it influences BBB function remains unclear. *In vivo* evidence suggests that the protective effect of sodium butyrate relates to its ability to stabilize BBB integrity, with sodium butyrate treated GF mice displaying reduced Evans blue extravasation in multiple brain regions and increased occludin levels (Braniste et al., 2014). In a model of transient focal cerebral ischemia sodium butyrate attenuated BBB permeability, suppressed NFκB-mediated production of MMP9, and prevented tight junction degradation (Wang et al., 2011). In a mouse model of TBI, decreased expression of occludin and Zonula Occludens-1 (ZO-1) was identified 24 h post-injury, a response that was ameliorated with sodium butyrate treatment, resulting in restoration of BBB integrity and beneficial neurological outcomes (Li et al., 2016). Whether these changes in barrier permeability induced by circulating SCFAs involve direct effects on the brain endothelium or indirect signaling by the enteroendocrine cells of the gut warrants further investigation.

Microbial metabolites can also influence immune responses in the periphery, which in turn affect BBB integrity through activation of TLRs. Lipoteichoic acid (LTA), a cell wall component of gram-positive bacteria and TLR2 agonist, activates peripheral immune responses and downstream signaling cascades, resulting in BBB dysfunction and behavioral abnormalities. In this manner, intra-peritoneal administration of *bacillus subtilis*-derived LTA increases pro-inflammatory IL-1β, IL-6, TNFα, and IFNγ cytokine expression in the circulation (Mayerhofer et al., 2017). These LTA-induced immunological alterations are accompanied by decreased claudin-5, occludin and tight junction protein-1 expression in the amygdala, highlighting the capacity of LTA to influence BBB composition through peripheral-CNS communication.

In addition to the regulation of BBB integrity by the microbiome, astonishing new findings now suggest that the microbiome impacts transport mechanisms across an intact barrier. In this manner, microbially-derived peptidoglycan (PGN) crosses the BBB, activating various PGN-recognition proteins (Pglrp1, Pglrp2, Pglrp3, Pglrp4), as well as TLR2 and the NOD-like receptors in the developing brain (Arentsen et al., 2017). Manipulation of the microbiome through GF and/or ABX-treated mice reduces CNS expression of the aforementioned recognition proteins, as well as the PGN transporter (PepT1). Interestingly, genetic deletion of Pglrp2 changes synapse-related gene expression and induces sex-dependent variations in social behavior, similar to that observed in microbially-perturbed mice (Arentsen et al., 2017). These findings challenge traditional view that microbial products only translocate into the brain in situations of compromised BBB integrity. This discovery of independent transit across the BBB and subsequent CNS immune signaling presents a novel axis for future investigation into the mechanism underlying microbiota-brain communication.

### Blood-cerebrospinal fluid-brain barrier (BCSFBB)

Diversification of the microbiota and their metabolites is a critical regulator of peripheral immune cell proliferation and activity (Dorrestein et al., 2014). In particular, the microbiome closely regulates the innate lymphoid cell populations of the gastro-intestinal tract (Gury-Benari et al., 2016) and these cells can, in turn, modulate T-cell populations residing in the lamina propia and mesenteric lymph nodes (Sonnenberg and Artis, 2015). T-cells circulate around the body and enter the brain via the choroid plexus (CP) (Engelhardt and Ransohoff, 2005), responsible for producing cerebrospinal fluid (CSF), whereby they contribute to neuro-inflammatory events and modulate microglial activity. Hence alterations in the commensal microbiome may be communicated to the brain via an altered immune status at the BCSFBB (Figure 1E).

The composition of immune cells populating the CP has important ramifications for neuro-inflammatory responses and brain health. The CP is populated with a subset of CD4^+^ memory T-cells that display T-cell receptor profiles unique to CNS-derived antigens. In aged mice these cells favor a Th2 response with elevated IL-4 and decreased type-2 IFNγ production that stimulates the CP epithelium to drive CCL11 production known to be deleterious to brain function (Villeda et al., 2011). Experimentally-induced proliferation of memory T cells restored the IL-4:IFNγ imbalance at the CP in these mice and prevented cognitive deficits (Baruch et al., 2013). Additional studies have now identified a CP-localized type-1 IFN response that drives deleterious aging. Importantly, aged mice supplemented with a type-1 IFN neutralizing antibody displayed reduced CCL11 expression, attenuated hippocampal glial reactivity, elevated BDNF production, and improved neurogenesis (Baruch et al., 2014). A dysregulated type-1 IFN signature has been observed in aging disorders, that appear to be regulated by the microbiome, including AD (Taylor et al., 2014; Minter et al., 2016a) and PD (Main et al., 2016) and it is now considered that a balance of type-1 and type-2 IFN signaling at the CP is required to impart healthy brain immunity and aging (Deczkowska et al., 2016).

Studies now suggest that intervening with “immune checkpoints” to re-correct immune cell phenotypes promotes clearance of pathogenic peptides (Aβ or α-syn) and cellular debris in neuro-degenerative disorders. Transient deletion of FoxP3^+^ T-regs triggers an IFNγ-mediated immune activation of the CP and results in recruitment of peripheral macrophages and repopulating T-regs, facilitating removal of Aβ plaques and restoring cognitive function in 5xFAD mice (Baruch et al., 2015). Similar beneficial IFNγ-dependent immune activity and Aβ clearance has also been demonstrated by programmed cell death protein-1 (PD-1) immune checkpoint blockade in the same animal model (Baruch et al., 2016). It is evident that re-programming, not dampening, of immune responses is critical in the treatment of neurodegenerative disorders. Considering perturbations in commensal microbial diversity alter peripheral T-cell compartments it will be critical to assess how these cell populations are affected at the CP immune barrier. Furthermore, it remains plausible that alterations in microbial diversity may trigger transient changes in T-cell phenotypes recruited to the CP that mimic the protective or deleterious phenotypes of CP-recruited cells observed in the aforementioned studies. Additional studies focusing on the BCSFBB in microbially-perturbed neurodegenerative disease models will provide much needed insight into how the commensal microbiome regulates brain immunity and identify novel therapeutic targets.

## Conclusion

Evidence presented from numerous studies now indicates the commensal microbiome exhibits fine control over host immunity that may have implications in developmental and neurodegenerative disorders. The precise mechanisms by which a perturbation in the host microbiome triggers alterations in peripheral immune compartments and neuro-inflammatory responses remains far from understood. We propose that investigations focusing on “immune barriers” of the brain, namely the BBB and BCSFBB, will provide important insights into how the microbiome communicates with the brain and we encourage future studies targeting the microbiome in neurodegenerative diseases to address these barriers (Figure 1). These studies may identify novel microbially-derived therapies to induce host immunological alterations and treat these disorders.

## Author contributions

BM and MM conceptualized, intellectualized, wrote, reviewed, and edited the manuscript.

### Conflict of interest statement

The authors declare that the research was conducted in the absence of any commercial or financial relationships that could be construed as a potential conflict of interest.

## Abbreviations

α-syn: Alpha-synuclein
ABX: Antibiotics
AD: Alzheimer's disease
APPSWE: Amyloid precursor protein (Swedish mutant)
Aβ: Amyloid-beta
BBB: Blood-brain barrier
BCSFBB: Blood-cerebrospinal fluid-brain barrier
BDNF: Brain-derived neurotrophic factor
CCL: Chemokine (C-C motif) ligand
CD: Cluster of differentiation
CNS: Central nervous system
CP: Choroid plexus
CSF: Cerebrospinal fluid
CXCL: Chemokine (C-X-C motif) ligand
DAMP: Damage-associated molecular pattern
DNA: Deoxyribonucleic acid
EAE: Experimental autoimmune encephalitis
FAD: Familial Alzheimer's disease
FoxP3: Forkhead box P3
GABA: Gamma-aminobutyric acid
GALT: Gut-associated lymphoid tissue
GF: Germ-free
GI: Gastrointestinal
HPA: Hypothalamic-pituitary axis
IFN: Interferon
IL: Interleukin
LPS: Lipopolysaccharide
LTA: Lipoteichoic acid
MCAO: Middle cerebral artery occlusion
MMP: Matrix metalloproteinase
MS: Multiple Sclerosis
NOD: Nucleotide-binding oligomerization domain
NFκB: Nuclear factor kappa B
PD: Parkinson's disease
PD-1: Programmed cell death protein-1
PGN: Peptidoglycan
PS1_ΔE9_: Presenilin-1 (exon 9 deletion)
PSA: Polysaccharide A
SCFA: Short-chain fatty acid
SPF: Specific pathogen-free
TBI: Traumatic brain injury
TLR: Toll-like receptor
TNF: Tumour necrosis factor
T-reg: T-regulatory cell
ZO-1: Zona occludens-1.
